# Diabetic kidney disease: Its current trends and future therapeutic perspectives

**DOI:** 10.1111/jdi.13121

**Published:** 2019-08-22

**Authors:** Daisuke Koya

**Affiliations:** ^1^ Department of Diabetology and Endocrinology Division of Anticipatory Molecular Food Science and Technology Kanazawa Medical University Ishikawa Japan

## Abstract

Diabetic kidney disease.

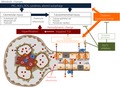

Diabetic kidney disease (DKD) is a leading cause of end‐stage renal disease (ESRD) worldwide and is induced by hyperglycemia, obesity, and high blood pressure^1^. Despite good control of blood glucose, blood pressure, and lipid levels^2^, the remaining risk for ESRD is still high, indicating an urgent need for new therapeutic strategies to prevent the development and progression of DKD. We and others have identified the underlying mechanism by which diabetes and hyperglycemia induce DKD, such as protein kinase C (PKC) activation, increased levels of cytokines, advanced glycation end products, oxidative stress, and altered stress responses^3^, ^4^, ^5^, ^6^, ^7^, ^8^. However, all drugs have failed to prevent the development and progression of DKD in human trials. Based on previous evidence, such as that from the RENAAL (Reduction of Endpoints in NIDDM with the Angiotensin II Antagonist Losartan) study and the IDNT (Irbesartan Diabetic Nephropathy Trial)^1^, renin‐angiotensin‐aldosterone (RAAS) inhibitors have been recognized as a specific drug for DKD. RAAS inhibitors definitely reduce albuminuria but fail to inhibit renal function decline, resulting in a decreased prevalence of albuminuric DKD and an increased prevalence of eGFR decline DKD^9^, ^10^.

In the past 4 years, sodium glucose cotransporter 2 inhibitors (SGLT2i) have been shown to have a high ability to intervene in the progression of DKD in the EMPA‐REG Outcome (*Empagliflozin*, Cardiovascular Outcomes, and Mortality in Type 2 Diabetes) study, CANagliflozin cardioVascular Assessment Study (CANVAS) program and DECLAIR TIMI 58 (Dapagliflozin Effect on CardiovascuLAR Events) study^11^. In patients with a past history of cardiovascular events, SGLT2 inhibitors reduced the renal endpoints by 44%. In patients without a past history of cardiovascular events, SGLT2 inhibitors reduced the renal endpoints by 46%. Moreover, Kadowaki *et al*.^12^ povided the first evidence in Asia that empagliflozin reduces the risk of the development or progression of DKD by 36%, the risk of progression to macroalbuminuria by 36%, and the composite risk of the doubling of serum creatinine and the initiation of renal‐replacement therapy or renal death by 52%. However, the renal endpoints have not been set as the primary outcomes but rather as the secondary outcomes, and therefore, these drugs are not recommended for DKD. Interestingly, Miyoshi *et al*.^13^ eported that even in type 2 diabetic patients with estimated glomerular filtration rate (eGFR) less than 60 mL/min/1.73 m^2^, two‐year treatment with SGLT2 inhibitors significantly preserved the eGFR compared to treatment with non‐SGLT2 inhibitors. Surprisingly, in the CREDENCE (The Canagliflozin and Renal Endpoints in Diabetes with Established Nephropathy Clinical Evaluation) study, the primary outcome was a composite of ESRD, the doubling of the serum creatinine level, and death from renal or cardiovascular disease. The canagliflozin treatment group reduced the primary outcome by 30% compared to the placebo group^14^.

It is important to know the underlying mechanisms by which SGLT2 inhibitors exert DKD protection. SGLT2 inhibitors reduce high levels of glucose, systolic blood pressure (SBP), body weight, uric acid, and ectopic fat, while they increase the levels of hematocrit and high density lipoprotein (HDL) cholesterol. Thus, SGLT2 inhibitors have systematic pleiotropic effects, thereby protecting the kidney from diabetic conditions. One of the other mechanisms is the normalization of altered tubuloglomerular feedback induced by diabetic conditions (Figure 1). This results in the normalization of diabetes‐induced glomerular hyperfiltration and higher intraglomerular pressure, which are well‐known accelerators of kidney injury. Recently, emerging evidence has focused on diabetes‐induced tubulointerstitial fibrosis, which is the end stage of kidney injury, resulting in the loss of renal function^7^. We also hypothesized that the direct toxicity of diabetes‐induced reabsorbed glucose and Na^+^ overload in the proximal tubular cells due to sodium–glucose cotransporter 2 could result in tubulointersitial fibrosis and nephron loss. We fed C57BL/6 mice a high‐fat diet (HFD) for 8 weeks and divided them into groups receiving the vehicle or ipragloflozin treatment for another 8 weeks^15^. Although mice with ipragliflozin treatment gained more weight and had higher glucose levels, the renal histology revealed that HFD‐fed mice displayed tubular vacuolation, dilatation and epithelial cell detachment, which were not observed in control mice; the administration of ipragliflozin ameliorated these alterations. In ultrastructural analysis, the tubular mitochondria of HFD‐fed mice exhibited significant damage, such as ballooning and the loss of cristae, and ipragliflozin treatment reversed the damage, restoring optic atrophy factor 1 and mitofusion 2 levels. SGLT2 inhibition could act directly on tubular cells and protect kidney tubular cells from mitochondrial damage due to metabolic insults regardless of blood glucose levels or the improvement in bodyweight reduction (Figure 1). SGLT2 inhibitors could contribute to fight against DKD development and progression in future.

**Figure 1:**
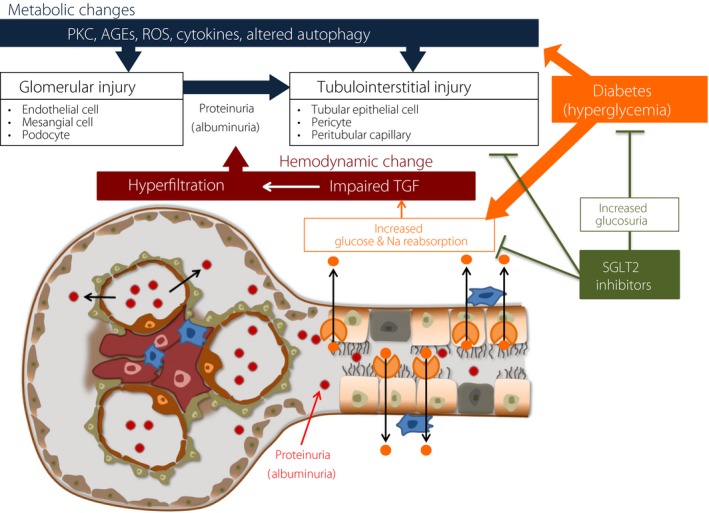
Reno‐protective effects of (sodium glucose cotransporter 2) SGLT2 inhibitors. Diabetes‐induced hyperglycemia, high blood pressure, high uric acid levels, and dyslipidemia causes PKC activation, oxidative stress, increased levels of cytokines, and altered autophagy in glomerular cells and proximal tubular epithelial cells in addition to hemodynamic disarrangement (altered tubuloglomerular feedback*;* TGF), resulting in glomerular and tubulointerstitial injury. After diabetic kidney disease (DKD) develops, it can progress to end‐stage renal disease, requiring renal‐replacement therapy or renal transplantation. Recent evidence has revealed that SGLT2 inhibitors not only systemically improve metabolic abnormalities but also reverse altered TGF and direct proximal tubulointerstitial injury. AGEs, advanced glycation end products; PKC, protein kinase C; ROS, reactive oxygen species; TGF, tubuloglomerular feedback.

## Disclosure

The author declares no conflict of interest.
